# Social media, nature, and life satisfaction: global evidence of the biophilia hypothesis

**DOI:** 10.1038/s41598-020-60902-w

**Published:** 2020-03-05

**Authors:** Chia-chen Chang, Gwyneth Jia Yi Cheng, Thi Phuong Le Nghiem, Xiao Ping Song, Rachel Rui Ying Oh, Daniel R. Richards, L. Roman Carrasco

**Affiliations:** 1 Department of Biological Sciences, National University of Singapore, 14 Science Drive 4, Singapore, 117543 Singapore; 2 Department of Architecture, National University of Singapore, 117566 Singapore, Singapore; 3 School of Biological Sciences, Centre for Biodiversity and Conservation Sciences, University of Queensland, 4072 Brisbane, Australia; 4 ETH Zurich, Singapore-ETH Centre, 1 Create Way, 138602 Singapore, Singapore

**Keywords:** Human behaviour, Psychology and behaviour

## Abstract

Humans may have evolved a need to connect with nature, and nature provides substantial cultural and social values to humans. However, quantifying the connection between humans and nature at a global scale remains challenging. We lack answers to fundamental questions: how do humans experience nature in different contexts (daily routines, fun activities, weddings, honeymoons, other celebrations, and vacations) and how do nature experiences differ across countries? We answer these questions by coupling social media and artificial intelligence using 31,534 social media photographs across 185 countries. We find that nature was more likely to appear in photographs taken during a fun activity, honeymoon, or vacation compared to photographs of daily routines. More importantly, the proportion of photographs with nature taken during fun activities is associated with national life satisfaction scores. This study provides global evidence of the biophilia hypothesis by showing a connection between humans and nature that contributes to life satisfaction and highlights how nature serves as background to many of our positive memories.

## Introduction

Ecosystems provide multiple benefits to humans, encompassing economic, ecological, cultural, and social values^1,2^. Despite these benefits, continuing environmental degradation has placed millions of animal and plant species under risk of extinction^3,4^. Removal and degradation of natural environments is expected to have negative consequences on human wellbeing^5^. This disparity between the overexploitation of natural resources and its importance to humans stems largely from the difficulty in integrating the value of nature’s benefits to people (“ecosystem services”) into policy^6^.

The value of ecosystem services is complex and multifaceted^6^. Although significant progress has been made in the economic and ecological valuation of ecosystem services, much less attention has been paid to cultural and social values, which are the most complex to capture^7^. Cultural ecosystem services are intangible benefits that people gain from experiencing nature^7,8^. The concept of “nature” is amorphous, so here we define nature as including biodiversity, ecosystems, living organisms, landscapes, and seascapes^5^. Nature provides an environmental space for cultural practices (including interacting with nature directly or using nature as background for other social activities) and yields various benefits^9^. These benefits include, among others, spiritual experiences, recreation, ecotourism, aesthetic appreciation, and further improved social cohesion and subjective wellbeing^5–9^.

Quantifying cultural ecosystem services is challenging as they represent immaterial benefits and the assessment involves untangling the reasons behind why people enjoy a particular space^7,10^. Collecting such information involves surveys or interviews that are resource-intensive and typically limited to small spatial scales^11,12^. Especially, how people experience nature in everyday lives (e.g., urban greenspace) and how people interact with nature under different contexts (e.g., relaxation, celebration, socialization, or daily routines) are particularly difficult to study in a large spatial scale. Recent breakthroughs in the study of cultural ecosystem services and understanding human-nature interactions have been possible through the use of social media. For instance, analyzing the user-defined “tags” of photographs can help understand the context under which the photograph was taken and potentially the self-reported emotional state of users. Analysis of social media photographs has been used, for instance, to study recreational^13^ and aesthetic qualities of natural areas^14^, preferences for nature-based activities in protected areas^15^, and associations between the use of outdoor space and happiness^16^. Despite these advances, global multi-country comparisons of cultural ecosystem services are lacking. Coupling social media with artificial intelligence for automated approaches in image recognition opens up unique opportunities to carry out large-scale studies of cultural ecosystem services to advance our understanding in human-nature relationships^17^.

One discipline that has studied the relationships between the experience of nature and human wellbeing is environmental psychology. According to the biophilia hypothesis (i.e., humanity’s innate tendency to connect with nature), humans largely relied on natural resources for survival and reproduction in human history, leading humans to evolve a tendency to prefer being close to nature through an emotional connection^18^. Psychological studies have demonstrated the capacity of nature to increase life satisfaction and improve attention restoration and stress recovery^19–21^. The psychological benefits gained from experiencing nature provide an important aspect of cultural ecosystem services^5^. People’s favorite places tend to have high restorative potential^10^. The locations where individuals can feel relaxed, forget their worries, and reflect on personal matters are often natural spaces^10^. We hypothesize that nature may play a role as a backdrop for key social contexts in a human’s life.

To test this hypothesis, we integrate both the fields of ecosystem services and environmental psychology to study how humans experience nature in various contexts, and how this relates to life satisfaction scores at a national level. Using the concept of cultural ecosystem services, we aim to analyze the links between nature (background), cultural practices (various contexts and activities), and benefits (cultural association between nature and positive social contexts and further life satisfaction). Based on the biophilia hypothesis and the capacity of nature for psychological restoration^19–21^, we hypothesize that humans tend to associate nature with positive social contexts, such as fun activities, celebrations, weddings, honeymoons, and vacations. In addition, we also investigate whether the relationship between nature experience and life satisfaction holds true at a cross-cultural level. We hypothesize that a nation with a stronger culture of experiencing nature would show higher life satisfaction as compared to other nations with a weaker culture in nature experience. We do this at an unprecedented global scale by leveraging on social media data and image recognition using machine learning algorithms.

We analyzed a total of 31,534 social media photographs uploaded on Flickr—a popular social media platform—using the Google Cloud Vision API. We used Flickr as the source of data because there are a large number of users (over 70 million users) and geotagged photographs (over 197 million)^13^. Flickr contains information about the location where many of the uploaded photographs were taken. These geotagged photographs allowed us to identify which country photographs were taken in. The photographs used in this study were geo-located across 185 countries, over a period of 11 years. We first assessed nature labels (i.e., image contents detected and generated by Google Cloud Vision API as nature-related labels) in photographs tagged by the users as “nature” and later checked the frequency of those labels within photographs tagged with specific contexts by users: people’s daily routines (as a baseline for comparisons with other contexts), fun activities, weddings, celebrations, honeymoons, and vacations. These social contexts were selected as they are likely to reflect people’s choice of favorite places when holding memorable social events/activities in their lives.

## Results

**Figure 1:**
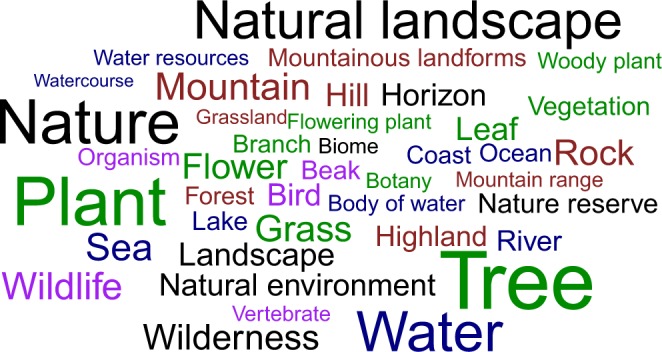
Word cloud showing the 40 most common nature labels detected by the image content analysis in 5,362 nature-tagged photographs. Word size is proportional to the frequency of occurrence. Nature labels were subsequently categorized into five different nature categories (color-coded, green: plants, brown: terrestrial landscapes, black: general terms, blue: water, purple: animals).

Analyzing the content of 5,362 photographs tagged by users as “nature”, we listed the most common nature labels identified by the image content analysis. These common nature labels covered from 7.3% to 40.2% of photographs (Fig. 1). These labels were subsequently categorized as: water, terrestrial landscapes, plants, animals, and nature in general terms (Fig. 1).

**Figure 2:**
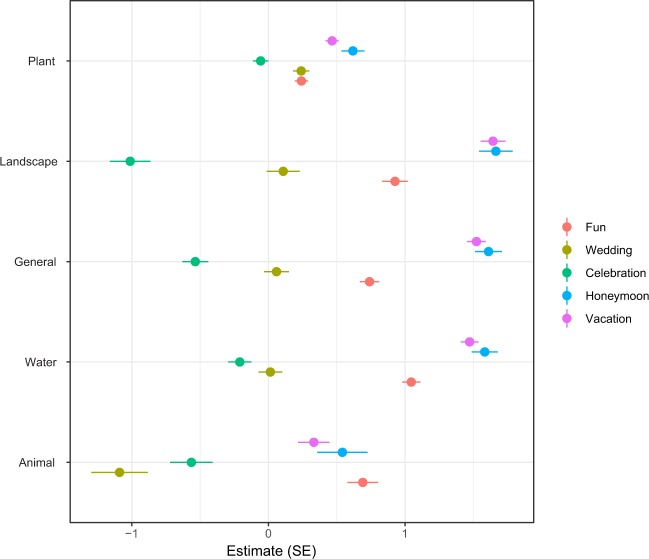
The relationship between social contexts and the presence of nature. The coefficient estimate (± SE) of the generalized linear mixed-effects models for each social context and nature category. A positive (negative) coefficient indicates a more (less) propensity for photographs to contain nature labels than the control photographs. Control photographs were used as the baseline (photographs tagged with “daily” or “routine”). Fun activity, honeymoon, and vacation photographs were more likely to contain nature labels as compared to daily routine photographs, for all categories of nature (Table S1). Celebration photographs were less likely to have nature labels than daily routine photographs, except for plants (Table S1).

**Figure 3:**
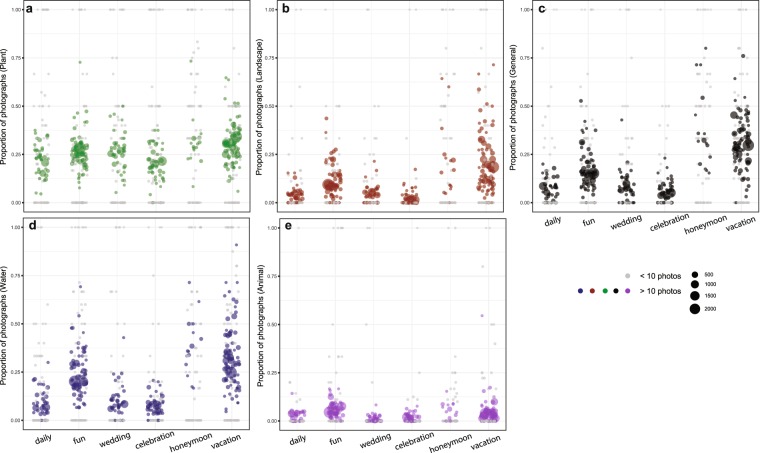
The proportion of photographs with nature labels identified with different nature categories (plants, terrestrial landscapes, general terms, water, animals) for each social context (daily routines, fun activities, weddings, celebrations, honeymoons, and vacations). Each point represents one country, and the size of points is proportional to the total number of photographs, and grey points represent the total number of photographs that are less than 10.

Comparing the frequencies of these nature labels identified in photographs tagged with various contexts by users (n = 26,172 photographs), we found that, across all five nature categories, photographs tagged with fun activities, honeymoons, and vacations were more likely to have nature labels identified in them than photographs tagged with daily routines (Figs. 2, 3, Table S1). Honeymoon and vacation photographs were more likely to have nature labels in them than fun activity photographs, with the exception of animals (Table S1). However, there was no difference between honeymoon photographs and vacation photographs in terms of the frequency of nature labels identified (Table S1). Celebration photographs were less likely to have nature labels than daily routine photographs, except for plants (Figs. 2, 3, Table S1). There was generally no significant difference between wedding photographs and daily routine photographs, except that wedding photographs were likely to have more plants and less animals (Figs. 2, 3, Table S1). This indicates that people tend to associate fun activities, honeymoons and vacations with nature, but not celebratory social events.

There was a wide variation in terms of how commonly nature appeared in photographs across countries (Table S2, Fig. 3). Nature commonly appeared in the photographs taken in some countries (e.g., for general nature terms: Iceland, Tanzania, Maldives, New Zealand, and Montenegro), but not in others (e.g., for general nature terms: Russia, Myanmar, China, Czech Republic, and Singapore).

**Figure 4:**
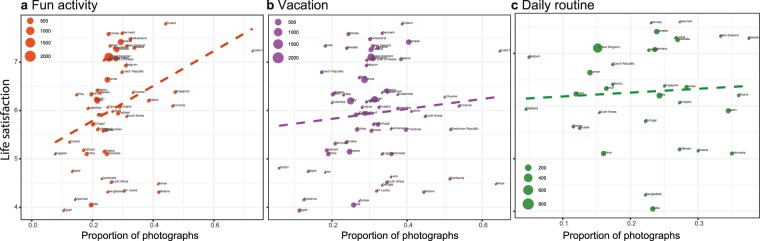
The relationship between national life satisfaction scores and the proportion of photographs with plant-related labels identified in three social contexts (**a** fun activity, **b** vacation, **c** daily routine). National life satisfaction was positively associated with the proportion of nature labels (plants) in fun activity photographs, but not associated in the context of vacations and daily routines. The size of the point is proportional to the number of photographs.

We found that, at a cross-national level, there was a positive association between the national life satisfaction score and the proportion of nature labels (plants) in the fun activity photographs (Fig. 4a, Table S3, Coefficient = 4.70 ± 1.29, t value = 3.64, unadjusted *p* value = 0.0006, FDR adjusted *p* value = 0.039). However, this relationship was not significant in the vacation photographs (Fig. 4b, Table S3, Coefficient = 1.95 ± 1.21, t value = 1.61, unadjusted *p* value = 0.113, FDR adjusted *p* value = 0.516), which may have been taken by a higher proportion of overseas tourists. This relationship was also not significant in daily routine photographs (Fig. 4c, Table S3, Coefficient = −1.95 ± 3.96, t value = −0.49, unadjusted *p* value = 0.626, FDR adjusted *p* value = 0.881). These results suggest that the context-dependent relationship between the national level of life satisfaction score and nature experience appears in the residents of the country.

## Discussion

Our results reveal that people are more likely to interact with nature in the context of fun activities, honeymoons, and vacations, suggesting an association between nature and these fun or relaxing moments. We also find that countries with more nature (plant-related) in fun activity photographs had higher life satisfaction, such as Costa Rica and Finland. These results, taken together, suggest the importance of nature in providing the background to positive social contexts, presumably fond memories, as well as in contributing to life satisfaction in communities worldwide.

A preference for natural environments during fun activities supports the biophilia hypothesis^18^. This biophilic relationship is more evident in the context of vacations and honeymoons, as both social contexts are intended to provide relaxation from daily routines and the possibly stressful period of organizing weddings or other celebratory events. This implies that humans not only associate nature with emotional happiness but also desire to experience nature probably because of experiences of awe, relaxation, and stress relief ^22,23^. For instance, visiting nature has been shown to improve cognitive ability, reduce stress, and lower the risk of depression^5,19,24^. These results further confirm the importance of nature for travel and tourism worldwide^25^, which not only provides economic value but also psychological and cultural values.

Landscape aesthetics as a cultural ecosystem service is particularly important given that the biophilic relationship is pervasive across cultures. Analyzing photographs allows us to understand what and when people want to capture as memories and share with other people. The high frequency of nature in photographs taken during fun activities and vacations implies the significance of nature in some of our fondest memories. For example, national parks in South Africa and marine sites in the UK provide cultural and social values by providing a place identity (a sense of place, such as “reliving childhood memories” and “I miss these sites when I have been away from them for a long time”)^26,27^. Similarly, the Satoyama landscape in Japan tends to be regarded as “home” for many Japanese people^28^. Some other famous natural landscapes have been identified as important cultural values to local communities, such as the Waikaraka Estuary in New Zealand^29^ and the Arafura-Timor seascape in Southeast Asia^30^. The human influence and loss of nature could potentially lead to the loss of these natural backgrounds to fond memories as well as diminish the cultural values of ecosystem services^30^.

In contrast, wedding photographs were not significantly different from daily routine photographs in terms of the presence of nature labels, and celebration photographs were generally less likely to have nature than daily routine photographs. This suggests that, unlike honeymoons or vacations, urban areas and closed settings (e.g. hotels) are chosen presumably for the convenience to organize social gatherings through high accessibility and to conform to traditional ceremonies^31^, and are thus prioritized over biophilic needs.

People vary in their connectedness to nature^32,33^. For example, some people spend time interacting with nature and perceive nature as an important component to their lives, but other people do not. We found that the frequency of nature that appeared in photographs varied widely across countries. This variation could be related to cultural and sociodemographic differences^34,35^. For example, it has been shown that Menominee Native Americans spend more time interacting with nature directly in their outdoor activities, as compared to European Americans^34^. Another comparative study also showed that Swiss participants preferred forests with high biodiversity, while Chinese participants did not show such preference^35^. The cultural variation in nature connectedness is important to be considered in the assessment and research in cultural ecosystem services.

Our study further reveals a positive relationship between life satisfaction and the presence of nature in fun activity photographs across multiple countries. Being correlational, these results could either point towards nature contributing to life satisfaction through fun memories, or to the tendency of people satisfied with their lives to spend time in a natural setting. Further research should focus on disentangling the cause and effect behind the observed patterns, as this could be an opportunity to design better programs for interacting with nature and improving human wellbeing. This result also points to the potentially synergistic effect of having social activities in the presence of nature. Different from the other contexts analyzed, fun activities are likely to be a social setting where people tend to interact with each other in a group. The combination of both social interaction and nature connection can be more rewarding than having either element alone^36–38^. Being related to both humans and nature is likely to contribute to our life satisfaction. For instance, it has been shown that in natural environments people tend to behave more altruistically and less selfishly, and that nature enhances social cohesion in communities and increases life satisfaction^23,39^. Interactions with nature, or within a natural backdrop, could strengthen social cohesion and improve life satisfaction.

Our analyses present several limitations. Although we know the country where the photograph was taken, we do not know whether it was taken by a local or a foreigner travelling to the country. Also, our focus on English tags assigned by Flickr users biased our results toward English-speaking nations and users. Further research could attempt to replicate our methods across multiple languages and photograph-sharing platforms. Although we performed verification checks to ensure that user-assigned tags led to the intended photographs (e.g. we excluded “proposal” as a tag for a special life event because it turned out to be ambiguous), some tags may lead to unrelated pictures, thus introducing noise to the analysis.

Integrating both the fields of cultural ecosystem services and environmental psychology through a photograph analysis at an unprecedented scale, we showed that people have a preference for nature in their fun activities, vacations, and honeymoons globally. Although our study represents only small steps in this line of inquiry, the findings suggest there is a whole underestimated dimension of the relationship between humans and nature through positive social contexts, presumably in the form of fond memories ultimately associated with life satisfaction. The main implication is that the loss of nature may mean more than losing quantifiable economic and ecological benefits; it could also mean losing the background to our fondest memories.

## Methods

### Choice of tags and nature labels

To select suitable nature elements that people associate with nature, we used “nature” as the tag, which is a self-reported keyword added by social media users when they upload to increase the photographs’ visibility. The common nature-related labels detected and generated by the Google image recognition API within the nature-tagged photographs were used as the nature labels in subsequent analyses.

We considered six contexts in this study. These were daily routines (as the baseline for comparisons), fun activities, weddings, celebrations, honeymoons, and vacations. Similarly, we used “tags” to identify these contexts. Daily routine related tags “daily” and “routine”, on separate searches, were used to retrieve *daily routine* photographs to be used as the baseline for comparisons. To identify general fun activities, we used the tags “fun” and “activity” on separate searches to retrieve the *fun activity* photographs. To investigate whether nature labels were more likely to be present in critical life events (weddings and honeymoons), we used wedding-related tags “wedding” and “marriage” to retrieve *wedding* photographs. The tag “honeymoon” was used solely for the *honeymoon* photographs. To distinguish between weddings and other types of celebrations as well as between honeymoons and other types of vacations, we also used the tag “celebration” to correspond to the *celebration* photographs, and vacation-related tags “vacation”, “holiday”, and “travel” to retrieve *vacation* photographs. Contexts and the tags used are summarized in Table S4.

### Image extraction and content detection

To extract photographs globally, we used Flickr’s public API to retrieve photographs with tags. We used the abovementioned 12 target tags, and retrieved photographs across 11 years, from 1^st^ of January 2008 to 31^st^ December 2018. As users varied in the number of photographs uploaded, we randomly selected one photograph from each Flickr user per returned tag search and therefore each photograph corresponds to an unique user in each tag search. We retrieved only photographs that users of Flickr had chosen to make publicly visible, by filtering the privacy setting. We also extracted all other tags that users added in the retrieved photographs to confirm that the retrieved photographs contained the target tags. Photographs without target tags were removed. To identify the geographical location of the photographs, we also extracted the GPS coordinates of the photographs and used the *revgeo* package with OpenStreetMap^40^ to identify the country of origin (n = 185).

To automatically detect the content within photographs, we used the Google Cloud Vision API through the RoogleVision package in R v3.5.3^41^. We used the *label detection* function to detect the content in a photograph. The Vision API can detect and generate various labels such as general objects, activities, locations, and products. We extracted a maximum of 15 labels from each photograph with a minimum confidence score of 0.5 (ranging from 0 to 1).

We performed a random manual check of 200 photographs (10 photographs across 20 countries) to verify the tags linked with the intended photographs, locations of photographs, and the accuracy of label detection. Among 200 photographs, all photographs showed correct contexts and countries, and captured nature content correctly for 91% of photographs (182/200) with the use of our nature labels.

### Statistical analyses

#### Association between the presence of natural labels and tags

We obtained 5,362 nature-tagged photographs. To understand what natural elements people may associate with nature, we first identified the common natural labels in the nature-tagged photographs. The Google Cloud Vision API detected and generated a total number of 2,942 labels, and we selected the 50 most frequently shown labels (each label appeared at least in 389 photographs among nature-tagged photographs). After filtering out irrelevant and ambiguous labels (i.e., adaptation, evening, green, morning, photography, reflection, sky, cloud, atmosphere, and atmospheric phenomenon), we grouped the nature-related labels into five nature categories: water, terrestrial landscapes, plants, animals, and nature in general terms (Table S5 with frequency). These natural labels were used as the labels to identify the presence of nature in the photographs with various contexts.

Photographs that were retrieved using the “celebration” tag may actually be wedding photographs and, similarly, the “vacation” tag may retrieve honeymoon photographs. To further refine the separation of wedding photographs from generic celebration photographs, we searched “wedding” tags in celebration-tagged photographs, and those photographs were then categorized as wedding photographs. Similarly, we searched “honeymoon” tags among vacation-tagged photographs and considered those photographs as honeymoon photographs. After the regrouping, some photographs that were tagged with multiple target tags (e.g., fun and holiday) were included in the sample of more than one contexts, as they may contain multiple contexts according to our definitions. In total, we obtained 26,172 photographs, and 3,781 of them were categorized into more than one contexts. We had 3,236 photographs classed as daily routine photographs, 8,589 photographs classed as fun activity photographs, 3,098 photographs classed as wedding photographs, 4,227 photographs classed as celebration photographs, 880 photographs classed as honeymoon photographs, and 10,129 photographs classed as vacation photographs. To evaluate the effect of including photographs in multiple contexts on the conclusions, a second analysis was run with the dataset after removing repeated photographs (n = 22,391, Table S6).

We performed generalized linear mixed-effects models with a binomial error structure. The presence or absence of certain nature categories (according to previously identified nature labels) was coded as a response variable (e.g., a photograph in which it was detected the presence of the nature label “tree” was considered as an instance of “plants” in the nature category, Table S5). The context was coded as the fixed effect, and country was considered as the random effect. The random effect for country attempted to account for national-level cultural differences and availability of natural space. The random effect for each country was extracted using the *ranef* function. We performed a total of four sets of analyses with different contexts as the baseline: 1) comparing fun activities, weddings, celebrations, honeymoons, and vacations against daily routines, 2) comparing weddings, celebrations, honeymoons, and vacations against fun activities, 3) comparing between weddings and celebrations, and 4) comparing honeymoons and vacations. We ran five models (for each nature category separately) in each set of analyses except for the natural category animal in 3) and 4) due to convergence failures. The *p* values were adjusted for multiple comparisons using the false discovery rate (FDR, with a total of 53 *p* values).

#### Association between life satisfaction and presence of natural labels in photographs

To investigate the association between the life satisfaction and proportion of photographs with the presence of nature at a cross-national level, we calculated the proportion of the photographs containing nature labels (for each nature category) in each context (i.e., daily routine, fun activity, wedding, celebration, honeymoon, and vacation) for each country. To ensure that each country is adequately represented, we removed countries that had less than 10 photographs for a given context.

We used life satisfaction in the Cantril Ladder scale (ranging from 0 to 10) with the average of survey responses from each country in 2017^42,43^. To control for the income of countries, we used GDP per capita based on purchasing power parities in 2017^42–44^. A total of 69 countries were used in the statistical analysis.

We ran linear regressions with life satisfaction as a response variable, and GDP per capita (to control for the relationship between wealth and life satisfaction), proportion of photographs with nature labels for each nature category, and the interaction between both variables were considered as the explanatory variables. We ran different models for different social contexts and each nature category was run separately. The *p* values were adjusted for multiple comparisons using the false discovery rate (with a total of 60 *p* values).

## Supplementary information

Supplementary table.

## Data Availability

All the photographs data can be retrieved using Flickr’s public API, and national life satisfaction and GDP data are available in *Our World in Data* (see ref. ^43^).
