# Supplementary material for: Social media, nature, and life satisfaction: global evidence of the biophilia hypothesis
Source: Sci Rep. 2020 Mar 5;10:4125. doi: 10.1038/s41598-020-60902-w (PMC7057999; doi:10.1038/s41598-020-60902-w)
Supplement: Supplementary file 1 — Supplementary table. [file 41598_2020_60902_MOESM1_ESM.docx]

**Social media, nature, and life satisfaction: global evidence of the biophilia hypothesis**

Chia-chen Chang^Γ,1,*^, Gwyneth Jia Yi Cheng^Γ,1^, Le Thi Phuong Nghiem^,1^, Xiao Ping Song^2,4^, Rachel Rui Ying Oh^3^, Daniel R Richards^4,*^, L Roman Carrasco^1*^

^Γ^Equal contribution.

^*^Emails for correspondence: dbschcc@nus.edu.sg; richards@arch.ethz.ch; dbsctlr@nus.edu.sg

^1^Department of Biological Sciences, National University of Singapore, 14 Science Drive 4, Singapore 117543.

^2^Department of Architecture, National University of Singapore, 117566, Singapore.

^3^School of Biological Sciences, Centre for Biodiversity and Conservation Sciences, University of Queensland, Brisbane, 4072, Australia.

^4^ETH Zurich, Singapore-ETH Centre, 1 Create Way, 138602, Singapore

This file includes:

Table S1 to S6

Table S1. Models results summary (generalized mixed effect models to test the relationship between nature and social contexts, with all data).

| Baseline | Nature category | Fixed effects | Estimate | Std.Error | z value | Pr(>\|z\|) | p_adjust |
| --- | --- | --- | --- | --- | --- | --- | --- |
| daily | water | (Intercept) | -2.253 | 0.091 | -24.777 | <2e-16 |  |
|  |  | groupb.fun_tag | 1.046 | 0.067 | 15.643 | <2e-16 | 0.000 |
|  |  | groupc1.wedding_tag | 0.014 | 0.087 | 0.163 | 0.871 | 0.887 |
|  |  | groupc2.celebration_tag | -0.210 | 0.085 | -2.471 | 0.014 | 0.017 |
|  |  | groupd1.honeymoon_tag | 1.584 | 0.096 | 16.577 | <2e-16 | 0.000 |
|  |  | groupd2.vacation_tag | 1.473 | 0.066 | 22.484 | <2e-16 | 0.000 |
| daily | landscape | (Intercept) | -3.253 | 0.119 | -27.376 | <2e-16 |  |
|  |  | groupb.fun_tag | 0.927 | 0.094 | 9.874 | <2e-16 | 0.000 |
|  |  | groupc1.wedding_tag | 0.109 | 0.121 | 0.899 | 0.369 | 0.399 |
|  |  | groupc2.celebration_tag | -1.012 | 0.148 | -6.844 | 0.000 | 0.000 |
|  |  | groupd1.honeymoon_tag | 1.665 | 0.123 | 13.547 | <2e-16 | 0.000 |
|  |  | groupd2.vacation_tag | 1.644 | 0.091 | 18.078 | <2e-16 | 0.000 |
| daily | plant | (Intercept) | -1.304 | 0.054 | -24.096 | <2e-16 |  |
|  |  | groupb.fun_tag | 0.242 | 0.050 | 4.873 | 0.000 | 0.000 |
|  |  | groupc1.wedding_tag | 0.240 | 0.060 | 4.031 | 0.000 | 0.000 |
|  |  | groupc2.celebration_tag | -0.057 | 0.057 | -0.992 | 0.321 | 0.362 |
|  |  | groupd1.honeymoon_tag | 0.619 | 0.084 | 7.346 | 0.000 | 0.000 |
|  |  | groupd2.vacation_tag | 0.466 | 0.049 | 9.608 | <2e-16 | 0.000 |
| daily | animal | (Intercept) | -3.569 | 0.132 | -26.945 | <2e-16 |  |
|  |  | groupb.fun_tag | 0.691 | 0.113 | 6.119 | 0.000 | 0.000 |
|  |  | groupc1.wedding_tag | -1.090 | 0.207 | -5.262 | 0.000 | 0.000 |
|  |  | groupc2.celebration_tag | -0.564 | 0.156 | -3.615 | 0.000 | 0.000 |
|  |  | groupd1.honeymoon_tag | 0.542 | 0.185 | 2.933 | 0.003 | 0.005 |
|  |  | groupd2.vacation_tag | 0.333 | 0.115 | 2.884 | 0.004 | 0.005 |
| daily | general | (Intercept) | -2.369 | 0.087 | -27.211 | <2e-16 |  |
|  |  | groupb.fun_tag | 0.740 | 0.071 | 10.412 | <2e-16 | 0.000 |
|  |  | groupc1.wedding_tag | 0.059 | 0.091 | 0.652 | 0.515 | 0.546 |
|  |  | groupc2.celebration_tag | -0.535 | 0.095 | -5.632 | 0.000 | 0.000 |
|  |  | groupd1.honeymoon_tag | 1.612 | 0.098 | 16.383 | <2e-16 | 0.000 |
|  |  | groupd2.vacation_tag | 1.523 | 0.069 | 22.202 | <2e-16 | 0.000 |
| fun | water | (Intercept) | -1.223 | 0.073 | -16.690 | <2e-16 |  |
|  |  | groupc1.wedding_tag | -1.030 | 0.067 | -15.280 | <2e-16 | 0.000 |
|  |  | groupc2.celebration_tag | -1.253 | 0.064 | -19.560 | <2e-16 | 0.000 |
|  |  | groupd1.honeymoon_tag | 0.545 | 0.078 | 7.000 | 0.000 | 0.000 |
|  |  | groupd2.vacation_tag | 0.432 | 0.035 | 12.480 | <2e-16 | 0.000 |
| fun | landscape | (Intercept) | -2.345 | 0.090 | -25.995 | <2e-16 |  |
|  |  | groupc1.wedding_tag | -0.819 | 0.091 | -8.984 | <2e-16 | 0.000 |
|  |  | groupc2.celebration_tag | -1.938 | 0.125 | -15.523 | <2e-16 | 0.000 |
|  |  | groupd1.honeymoon_tag | 0.749 | 0.094 | 7.986 | 0.000 | 0.000 |
|  |  | groupd2.vacation_tag | 0.722 | 0.044 | 16.362 | <2e-16 | 0.000 |
| fun | plant | (Intercept) | -1.088 | 0.043 | -25.213 | <2e-16 |  |
|  |  | groupc1.wedding_tag | 0.001 | 0.048 | 0.031 | 0.975 | 0.975 |
|  |  | groupc2.celebration_tag | -0.299 | 0.045 | -6.598 | 0.000 | 0.000 |
|  |  | groupd1.honeymoon_tag | 0.386 | 0.077 | 5.040 | 0.000 | 0.000 |
|  |  | groupd2.vacation_tag | 0.232 | 0.033 | 6.939 | 0.000 | 0.000 |
| fun | animal | (Intercept) | -2.855 | 0.095 | -29.933 | <2e-16 |  |
|  |  | groupc1.wedding_tag | -1.780 | 0.186 | -9.598 | <2e-16 | 0.000 |
|  |  | groupc2.celebration_tag | -1.251 | 0.126 | -9.952 | <2e-16 | 0.000 |
|  |  | groupd1.honeymoon_tag | -0.147 | 0.160 | -0.919 | 0.358 | 0.395 |
|  |  | groupd2.vacation_tag | -0.364 | 0.069 | -5.267 | 0.000 | 0.000 |
| fun | general | (Intercept) | -1.639 | 0.066 | -24.970 | <2e-16 |  |
|  |  | groupc1.wedding_tag | -0.677 | 0.071 | -9.580 | <2e-16 | 0.000 |
|  |  | groupc2.celebration_tag | -1.274 | 0.076 | -16.860 | <2e-16 | 0.000 |
|  |  | groupd1.honeymoon_tag | 0.884 | 0.080 | 11.080 | <2e-16 | 0.000 |
|  |  | groupd2.vacation_tag | 0.789 | 0.037 | 21.080 | <2e-16 | 0.000 |
| wedding | water | (Intercept) | -2.242 | 0.088 | -25.435 | <2e-16 |  |
|  |  | groupc2.celebration_tag | -0.242 | 0.086 | -2.815 | 0.005 | 0.006 |
| wedding | landscape | (Intercept) | -3.014 | 0.123 | -24.548 | <2e-16 |  |
|  |  | groupc2.celebration_tag | -1.097 | 0.147 | -7.468 | 0.000 | 0.000 |
| wedding | plant | (Intercept) | -1.060 | 0.045 | -23.714 | <2e-16 |  |
|  |  | groupc2.celebration_tag | -0.268 | 0.058 | -4.621 | 0.000 | 0.000 |
| wedding | general | (Intercept) | -2.336 | 0.086 | -27.036 | <2e-16 |  |
|  |  | groupc2.celebration_tag | -0.579 | 0.095 | -6.069 | 0.000 | 0.000 |
| honeymoon | water | (Intercept) | -0.657 | 0.101 | -6.502 | 0.000 |  |
|  |  | groupd2.vacation_tag | -0.137 | 0.076 | -1.792 | 0.073 | 0.086 |
| honeymoon | landscape | (Intercept) | -1.603 | 0.126 | -12.687 | <2e-16 |  |
|  |  | groupd2.vacation_tag | -0.039 | 0.091 | -0.429 | 0.668 | 0.694 |
| honeymoon | plant | (Intercept) | -0.697 | 0.080 | -8.680 | <2e-16 |  |
|  |  | groupd2.vacation_tag | -0.156 | 0.076 | -2.070 | 0.039 | 0.046 |
| honeymoon | general | (Intercept) | -0.732 | 0.096 | -7.637 | 0.000 |  |
|  |  | groupd2.vacation_tag | -0.114 | 0.077 | -1.480 | 0.139 | 0.160 |

Table S2. The random effects for each country for all nature categories.

| Country | General | Landscape | Water | Plant | Animal | No. of photos |
| --- | --- | --- | --- | --- | --- | --- |
| Iceland | 1.633 | 1.892 | 0.993 | -0.295 | -0.509 | 171 |
| Tanzania | 1.124 | 0.091 | 0.811 | 0.125 | 1.193 | 30 |
| Maldives | 1.107 | -0.858 | 1.482 | 0.108 | -0.090 | 59 |
| New Zealand | 0.736 | 1.415 | 0.519 | 0.176 | 0.095 | 263 |
| Montenegro | 0.677 | 1.723 | 0.829 | 0.015 | 0.077 | 20 |
| Panama | 0.642 | -0.287 | 0.442 | 0.139 | -0.107 | 7 |
| Zambia | 0.601 | -0.034 | 0.440 | -0.030 | 1.269 | 9 |
| Egypt | 0.597 | 0.018 | -0.249 | -0.564 | 1.250 | 109 |
| Sudan | 0.597 | 1.216 | -0.276 | 0.139 | -0.040 | 3 |
| Peru | 0.554 | 1.444 | -1.069 | -0.038 | 0.029 | 86 |
| Malaysia | 0.539 | -0.192 | 0.593 | -0.050 | -0.055 | 192 |
| Fiji | 0.522 | -0.100 | 1.054 | 0.145 | -0.134 | 9 |
| Romania | 0.521 | 0.714 | -0.462 | 0.441 | -0.211 | 85 |
| Faroe Islands | 0.513 | 1.133 | 0.296 | -0.048 | -0.172 | 10 |
| Cape Verde | 0.511 | 0.031 | 0.881 | -0.112 | -0.098 | 6 |
| Namibia | 0.482 | 0.838 | -0.703 | 0.066 | 0.600 | 10 |
| Colombia | 0.473 | -0.028 | 0.301 | 0.080 | -0.421 | 51 |
| Norway | 0.471 | 0.933 | 0.327 | -0.105 | 0.012 | 231 |
| Morocco | 0.465 | 0.026 | -0.764 | -0.198 | 0.066 | 73 |
| Estonia | 0.455 | 0.123 | 0.092 | -0.016 | -0.293 | 24 |
| Slovenia | 0.452 | 0.352 | 0.111 | 0.089 | 0.655 | 23 |
| Costa Rica | 0.450 | 0.545 | 0.639 | 0.494 | -0.149 | 39 |
| Angola | 0.450 | -0.110 | -0.163 | -0.035 | -0.028 | 2 |
| Mongolia | 0.434 | 0.592 | -0.105 | 0.027 | -0.082 | 6 |
| Laos | 0.422 | -0.087 | -0.045 | 0.113 | -0.379 | 33 |
| Venezuela | 0.415 | -0.335 | 0.473 | -0.190 | -0.299 | 27 |
| South Africa | 0.408 | 0.748 | 0.283 | 0.077 | 0.964 | 120 |
| Georgia | 0.406 | 1.438 | -0.510 | -0.082 | 0.143 | 20 |
| Saint Lucia | 0.404 | -0.693 | 1.199 | -0.062 | -0.024 | 27 |
| Tunisia | 0.384 | -0.404 | -0.160 | 0.036 | -0.254 | 19 |
| Greece | 0.370 | 0.744 | 0.954 | -0.103 | -0.648 | 479 |
| Cook Islands | 0.348 | -0.205 | 1.556 | 0.121 | -0.168 | 13 |
| Bolivia | 0.345 | 0.496 | -0.506 | 0.059 | 0.277 | 8 |
| Croatia | 0.342 | 0.189 | 1.134 | -0.181 | 0.001 | 131 |
| Jordan | 0.334 | 0.996 | -0.982 | -0.221 | -0.317 | 26 |
| Lebanon | 0.312 | 0.287 | 0.348 | 0.047 | -0.071 | 5 |
| Portugal | 0.307 | 0.514 | 0.733 | -0.036 | 0.070 | 339 |
| Seychelles | 0.297 | 0.060 | 0.707 | 0.082 | 0.875 | 12 |
| Kenya | 0.295 | -0.009 | -0.409 | 0.257 | 1.537 | 29 |
| Argentina | 0.290 | 0.266 | -0.022 | -0.029 | 0.409 | 93 |
| Bosnia and Herzegovina | 0.285 | 1.099 | -0.194 | 0.136 | 0.544 | 14 |
| Lesotho | 0.271 | 0.549 | -0.042 | -0.017 | -0.004 | 1 |
| Guinea | 0.271 | -0.024 | -0.042 | 0.050 | -0.004 | 1 |
| Switzerland | 0.254 | 1.271 | -0.291 | 0.106 | 0.225 | 220 |
| Libya | 0.253 | -0.029 | -0.073 | 0.039 | 0.848 | 2 |
| Papua New Guinea | 0.226 | 0.495 | 0.277 | 0.036 | -0.030 | 2 |
| Australia | 0.210 | 0.108 | 0.463 | 0.058 | 0.153 | 752 |
| Tonga | 0.201 | 0.449 | 0.280 | -0.020 | 0.421 | 1 |
| Nigeria | 0.199 | -0.247 | -0.135 | -0.029 | -0.121 | 11 |
| Greenland | 0.190 | 0.965 | 0.201 | 0.027 | -0.086 | 6 |
| Chile | 0.180 | 1.093 | 0.007 | -0.161 | 0.236 | 70 |
| Bermuda | 0.179 | 0.153 | -0.124 | 0.083 | 0.320 | 6 |
| Bahamas | 0.176 | -0.761 | 0.586 | -0.115 | 0.414 | 22 |
| Cyprus | 0.158 | 0.141 | -0.043 | 0.064 | -0.220 | 52 |
| Honduras | 0.156 | -0.310 | 0.118 | 0.059 | -0.115 | 8 |
| Belize | 0.152 | 0.388 | 0.386 | 0.340 | 0.625 | 21 |
| Syria | 0.148 | -0.602 | -0.822 | -0.096 | -0.183 | 13 |
| Ukraine | 0.141 | 0.673 | -0.384 | 0.463 | 0.285 | 111 |
| Madagascar | 0.135 | 0.097 | 0.659 | -0.045 | -0.113 | 6 |
| San Marino | 0.108 | 0.333 | -0.239 | -0.040 | -0.033 | 2 |
| Saint Kitts and Nevis | 0.108 | -0.170 | -0.239 | 0.026 | -0.033 | 2 |
| Anguilla | 0.103 | -0.172 | 0.501 | -0.043 | -0.036 | 2 |
| Ecuador | 0.099 | 0.350 | 0.004 | 0.056 | 0.121 | 19 |
| Mauritius | 0.086 | -0.441 | 0.989 | 0.060 | 0.793 | 40 |
| Oman | 0.080 | 0.810 | 0.388 | 0.007 | -0.007 | 25 |
| Armenia | 0.065 | 0.307 | -0.312 | -0.031 | -0.060 | 6 |
| South Korea | 0.064 | 0.066 | -0.224 | 0.213 | 0.083 | 75 |
| Vanuatu | 0.063 | 0.025 | 0.033 | 0.127 | -0.104 | 7 |
| Samoa | 0.061 | -0.208 | -0.308 | -0.057 | -0.055 | 3 |
| Canada | 0.053 | 0.582 | -0.134 | 0.230 | -0.022 | 1114 |
| Albania | 0.052 | 0.401 | 0.576 | -0.074 | -0.102 | 8 |
| Zimbabwe | 0.033 | -0.225 | -0.341 | 0.123 | 0.744 | 4 |
| Brazil | 0.033 | -0.044 | 0.605 | -0.227 | -0.575 | 355 |
| Mexico | 0.029 | 0.044 | 0.228 | 0.034 | 0.076 | 316 |
| Nicaragua | 0.029 | -0.007 | 0.780 | 0.043 | -0.246 | 20 |
| Thailand | 0.027 | -0.341 | 0.151 | -0.058 | -0.728 | 450 |
| Sri Lanka | 0.020 | -0.587 | 0.218 | 0.073 | 0.291 | 68 |
| British Virgin Islands | 0.020 | 0.941 | 0.984 | -0.137 | -0.238 | 15 |
| Indonesia | 0.020 | 0.119 | 0.114 | 0.118 | -0.131 | 314 |
| Taiwan | 0.008 | 0.205 | -0.386 | -0.095 | 0.067 | 146 |
| Spain | 0.000 | 0.395 | 0.029 | -0.076 | -0.458 | 1068 |
| Luxembourg | -0.001 | 0.332 | -0.331 | 0.213 | -0.131 | 9 |
| South Sudan | -0.016 | -0.008 | -0.034 | 0.054 | -0.007 | 1 |
| Eritrea | -0.016 | -0.008 | -0.034 | -0.014 | -0.007 | 1 |
| Guyana | -0.016 | -0.008 | -0.034 | -0.014 | -0.007 | 1 |
| Kosovo | -0.016 | -0.008 | -0.034 | -0.014 | -0.007 | 1 |
| Uganda | -0.018 | -0.269 | -0.044 | 0.043 | -0.070 | 5 |
| Ireland | -0.019 | 0.546 | -0.284 | 0.087 | -0.180 | 230 |
| Malta | -0.021 | -0.239 | 0.519 | -0.151 | 0.028 | 53 |
| Pakistan | -0.021 | -0.037 | -0.301 | -0.189 | 0.455 | 40 |
| Afghanistan | -0.026 | -0.022 | 0.390 | 0.053 | -0.012 | 1 |
| Guinea-Bissau | -0.026 | -0.022 | -0.041 | 0.053 | -0.012 | 1 |
| United States of America | -0.027 | 0.128 | -0.213 | 0.112 | 0.017 | 7338 |
| Denmark | -0.030 | -0.513 | -0.292 | 0.087 | -0.060 | 118 |
| Belarus | -0.043 | -0.068 | -0.985 | 0.036 | 0.392 | 24 |
| São Tomé and Príncipe | -0.048 | -0.050 | -0.096 | 0.050 | -0.023 | 1 |
| Yemen | -0.048 | -0.050 | -0.096 | -0.017 | -0.023 | 1 |
| Iraq | -0.050 | -0.042 | -0.080 | 0.038 | -0.024 | 2 |
| Guernsey | -0.053 | -0.047 | -0.081 | -0.034 | -0.008 | 2 |
| Sweden | -0.060 | -0.271 | 0.148 | -0.050 | -0.293 | 187 |
| Bahrain | -0.063 | -0.058 | 0.277 | -0.031 | -0.030 | 2 |
| El Salvador | -0.070 | -0.275 | -0.392 | -0.069 | -0.214 | 15 |
| The Gambia | -0.073 | -0.072 | -0.132 | -0.034 | 0.405 | 2 |
| Rwanda | -0.077 | -0.065 | -0.153 | -0.044 | -0.037 | 3 |
| Cayman Islands | -0.078 | -0.319 | -0.127 | 0.097 | -0.081 | 5 |
| United Arab Emirates | -0.083 | -1.053 | -0.115 | -0.031 | -0.466 | 89 |
| Falkland Islands | -0.086 | -0.091 | -0.129 | 0.047 | -0.017 | 1 |
| Comoros | -0.086 | -0.091 | -0.129 | -0.020 | -0.017 | 1 |
| Paraguay | -0.086 | -0.091 | -0.129 | -0.020 | -0.017 | 1 |
| Moldova | -0.091 | -0.076 | -0.145 | -0.064 | -0.019 | 4 |
| Montserrat | -0.092 | -0.093 | -0.139 | -0.023 | -0.020 | 1 |
| Poland | -0.097 | -0.048 | -0.235 | 0.255 | -0.014 | 198 |
| Gibraltar | -0.098 | -0.339 | -0.488 | -0.043 | 1.071 | 6 |
| Burkina Faso | -0.099 | -0.084 | -0.185 | 0.204 | -0.048 | 4 |
| Dominica | -0.100 | -0.098 | 0.241 | -0.034 | 0.410 | 2 |
| Andorra | -0.106 | 0.424 | -0.206 | -0.048 | -0.052 | 3 |
| Togo | -0.106 | -0.103 | -0.206 | -0.048 | -0.052 | 3 |
| Philippines | -0.107 | -0.364 | 0.389 | 0.229 | -0.685 | 181 |
| East Timor | -0.108 | 0.434 | 0.550 | 0.073 | -0.053 | 4 |
| Haiti | -0.109 | 0.420 | 0.233 | -0.035 | -0.028 | 2 |
| Guatemala | -0.111 | -0.139 | -0.383 | -0.021 | -0.374 | 36 |
| Somalia | -0.113 | -0.105 | 0.579 | -0.047 | -0.030 | 3 |
| Uruguay | -0.128 | -0.304 | 0.688 | -0.058 | 0.139 | 19 |
| Turks and Caicos Islands | -0.129 | -0.134 | 0.548 | 0.096 | -0.039 | 2 |
| Djibouti | -0.129 | -0.134 | -0.210 | -0.037 | -0.039 | 2 |
| Cambodia | -0.130 | 0.186 | -0.345 | 0.286 | -0.572 | 62 |
| Turkmenistan | -0.133 | -0.131 | -0.196 | -0.054 | -0.025 | 3 |
| The Netherlands | -0.136 | -0.863 | 0.144 | 0.011 | 0.659 | 459 |
| Macedonia | -0.137 | -0.134 | -0.241 | -0.064 | -0.060 | 4 |
| Algeria | -0.139 | 0.435 | 0.053 | -0.059 | -0.114 | 7 |
| Jersey | -0.141 | -0.141 | -0.235 | -0.051 | -0.046 | 3 |
| Swaziland | -0.141 | -0.141 | -0.235 | -0.051 | -0.046 | 3 |
| Trinidad and Tobago | -0.147 | -0.145 | 0.425 | -0.064 | -0.073 | 4 |
| Antigua and Barbuda | -0.150 | -0.379 | 0.066 | 0.062 | 0.312 | 7 |
| Turkey | -0.153 | -0.073 | -0.289 | -0.081 | -0.111 | 252 |
| Azerbaijan | -0.154 | 0.356 | -0.227 | -0.070 | -0.029 | 4 |
| Saudi Arabia | -0.157 | -0.168 | -0.294 | -0.182 | -0.219 | 16 |
| Botswana | -0.161 | 0.409 | 0.033 | 0.002 | 1.696 | 7 |
| Mozambique | -0.162 | -0.157 | 0.101 | 0.002 | -0.057 | 4 |
| Kuwait | -0.171 | -0.157 | -0.320 | -0.025 | 0.321 | 6 |
| Senegal | -0.176 | -0.176 | -0.263 | -0.053 | -0.039 | 3 |
| Kyrgyzstan | -0.184 | -0.177 | -0.291 | 0.047 | -0.053 | 5 |
| United Kingdom | -0.195 | 0.126 | -0.164 | -0.058 | 0.271 | 3374 |
| Benin | -0.201 | -0.208 | -0.308 | 0.009 | -0.055 | 3 |
| Italy | -0.212 | 0.366 | 0.127 | -0.223 | -0.353 | 1442 |
| Palestinian Territory | -0.213 | -0.214 | -0.329 | -0.070 | -0.061 | 4 |
| Brunei Darussalam | -0.217 | -0.217 | 0.606 | -0.083 | 0.317 | 5 |
| Uzbekistan | -0.217 | -0.428 | -0.622 | -0.129 | -0.133 | 12 |
| Bangladesh | -0.220 | -0.567 | -0.228 | -0.242 | -0.588 | 93 |
| Bulgaria | -0.224 | 0.212 | -0.175 | -0.038 | -0.216 | 52 |
| Germany | -0.239 | -0.172 | -0.445 | 0.092 | 0.343 | 814 |
| Austria | -0.239 | 1.140 | -0.880 | 0.184 | -0.343 | 198 |
| Dominican Republic | -0.242 | -0.823 | 1.219 | 0.320 | -0.252 | 51 |
| Mali | -0.244 | -0.246 | -0.354 | -0.072 | -0.055 | 4 |
| Iran | -0.252 | -0.259 | -0.568 | -0.300 | -0.348 | 36 |
| Isle of Man | -0.260 | -0.473 | 0.315 | -0.057 | -0.165 | 11 |
| Kazakhstan | -0.272 | -0.267 | -0.064 | 0.091 | -0.065 | 6 |
| Finland | -0.280 | -0.117 | 0.062 | 0.299 | -0.121 | 104 |
| Japan | -0.290 | -0.308 | -0.596 | 0.090 | -0.216 | 451 |
| India | -0.299 | -0.728 | 0.054 | -0.201 | -0.066 | 781 |
| Nepal | -0.302 | 0.502 | -1.228 | -0.349 | -0.546 | 59 |
| Israel | -0.311 | -0.628 | 0.087 | -0.268 | -0.247 | 87 |
| Grenada | -0.334 | -0.338 | 0.100 | 0.082 | -0.113 | 6 |
| France | -0.353 | -0.254 | -0.033 | -0.045 | -0.004 | 1258 |
| Serbia | -0.353 | -0.338 | -0.348 | -0.061 | 0.052 | 29 |
| Tajikistan | -0.392 | -0.389 | -0.599 | -0.113 | -0.153 | 11 |
| Barbados | -0.393 | -0.587 | 0.973 | 0.073 | -0.155 | 13 |
| Qatar | -0.415 | -0.581 | -0.067 | -0.266 | 0.033 | 25 |
| Suriname | -0.419 | -0.410 | 0.506 | -0.035 | -0.124 | 10 |
| Ghana | -0.434 | 0.356 | 0.196 | -0.049 | -0.135 | 11 |
| Bhutan | -0.439 | 0.338 | -0.629 | 0.082 | -0.138 | 10 |
| Belgium | -0.508 | -0.327 | -0.315 | 0.006 | 0.392 | 204 |
| Lithuania | -0.541 | -0.537 | -0.403 | 0.265 | -0.343 | 32 |
| Hungary | -0.564 | -0.562 | 0.036 | 0.160 | -0.208 | 126 |
| Ethiopia | -0.572 | -0.189 | -0.819 | -0.191 | 0.147 | 17 |
| Vietnam | -0.583 | -0.661 | -0.185 | -0.129 | -0.558 | 215 |
| North Korea | -0.596 | -0.573 | -0.358 | -0.113 | -0.226 | 20 |
| Slovakia | -0.638 | -0.130 | -0.407 | 0.050 | 0.180 | 42 |
| Latvia | -0.649 | -0.519 | -0.604 | 0.140 | -0.200 | 49 |
| Cuba | -0.665 | -1.098 | 0.310 | -0.185 | -0.264 | 120 |
| Singapore | -0.928 | -0.769 | -0.409 | 0.320 | 0.502 | 179 |
| Czech Republic | -0.953 | -0.594 | -0.662 | -0.280 | -0.048 | 155 |
| China | -0.959 | -0.778 | -0.568 | -0.305 | -0.357 | 468 |
| Myanmar | -1.068 | -1.293 | -0.315 | -0.194 | -0.578 | 63 |
| Russia | -1.080 | -0.299 | -0.362 | 0.062 | 0.197 | 254 |

Table S3. Models results summary (linear regressions to associate the proportion of photographs with nature and life satisfaction in a cross-nation level).

| Context | Nature category | Variables | Estimate | Std. Error | t-value | Pr(>\|t\|) | p_adjust |
| --- | --- | --- | --- | --- | --- | --- | --- |
| daily | water | (Intercept) | 4.4040 | 0.4241 | 10.3840 | 0.0000 |  |
|  |  | GDP | 0.0001 | 0.0000 | 5.4340 | 0.0000 |  |
|  |  | water_prop | 4.1200 | 3.0640 | 1.3450 | 0.1895 | 0.5414 |
|  |  | GDP:water_prop | -0.0002 | 0.0001 | -2.6460 | 0.0132 | 0.1584 |
| daily | landscape | (Intercept) | 4.9990 | 0.3274 | 15.2700 | 0.0000 |  |
|  |  | GDP | 0.0000 | 0.0000 | 4.6130 | 0.0001 |  |
|  |  | landscape_prop | 0.4508 | 8.5820 | 0.0530 | 0.9580 | 0.9796 |
|  |  | GDP:landscape_prop | 0.0001 | 0.0002 | 0.3030 | 0.7640 | 0.9118 |
| daily | plant | (Intercept) | 5.4680 | 0.9371 | 5.8350 | 0.0000 |  |
|  |  | GDP | 0.0000 | 0.0000 | 0.8220 | 0.4180 |  |
|  |  | plant_prop | -1.9470 | 3.9550 | -0.4920 | 0.6260 | 0.8809 |
|  |  | GDP:plant_prop | 0.0001 | 0.0001 | 0.4650 | 0.6460 | 0.8809 |
| daily | animal | (Intercept) | 5.0710 | 0.2911 | 17.4180 | <2e-16 |  |
|  |  | GDP | 0.0000 | 0.0000 | 4.6910 | 0.0001 |  |
|  |  | animal_prop | -14.3600 | 13.7900 | -1.0410 | 0.3070 | 0.6750 |
|  |  | GDP:animal_prop | 0.0006 | 0.0004 | 1.6240 | 0.1160 | 0.5160 |
| daily | general | (Intercept) | 5.3910 | 0.3569 | 15.1050 | 0.0000 |  |
|  |  | GDP | 0.0000 | 0.0000 | 3.0480 | 0.0050 |  |
|  |  | general_prop | -8.3520 | 4.3230 | -1.9320 | 0.0635 | 0.5010 |
|  |  | GDP:general_prop | 0.0003 | 0.0001 | 2.3780 | 0.0245 | 0.2446 |
| fun | water | (Intercept) | 5.0460 | 0.4640 | 10.8750 | 0.0000 |  |
|  |  | GDP | 0.0000 | 0.0000 | 1.9410 | 0.0580 |  |
|  |  | water_prop | -1.4180 | 1.8790 | -0.7550 | 0.4540 | 0.8012 |
|  |  | GDP:water_prop | 0.0001 | 0.0001 | 1.0160 | 0.3150 | 0.6750 |
| fun | landscape | (Intercept) | 4.5680 | 0.2464 | 18.5410 | <2e-16 |  |
|  |  | GDP | 0.0000 | 0.0000 | 5.2070 | 0.0000 |  |
|  |  | landscape_prop | 2.2450 | 2.1570 | 1.0410 | 0.3030 | 0.6750 |
|  |  | GDP:landscape_prop | 0.0000 | 0.0001 | 0.1940 | 0.8470 | 0.9240 |
| fun | plant | (Intercept) | 3.4800 | 0.3866 | 9.0010 | 0.0000 |  |
|  |  | GDP | 0.0001 | 0.0000 | 5.4900 | 0.0000 |  |
|  |  | plant_prop | 4.7020 | 1.2920 | 3.6390 | 0.0006 | 0.0389 |
|  |  | GDP:plant_prop | -0.0001 | 0.0000 | -2.8160 | 0.0069 | 0.1362 |
| fun | animal | (Intercept) | 4.7980 | 0.2603 | 18.4300 | <2e-16 |  |
|  |  | GDP | 0.0000 | 0.0000 | 4.6990 | 0.0000 |  |
|  |  | animal_prop | -0.9076 | 3.7190 | -0.2440 | 0.8080 | 0.9167 |
|  |  | GDP:animal_prop | 0.0000 | 0.0001 | 0.1150 | 0.9090 | 0.9614 |
| fun | general | (Intercept) | 4.9870 | 0.3291 | 15.1550 | <2e-16 |  |
|  |  | GDP | 0.0000 | 0.0000 | 2.4540 | 0.0177 |  |
|  |  | general_prop | -1.8910 | 1.9550 | -0.9670 | 0.3381 | 0.6995 |
|  |  | GDP:general_prop | 0.0001 | 0.0001 | 1.5760 | 0.1213 | 0.5160 |
| wedding | water | (Intercept) | 4.5030 | 0.3626 | 12.4190 | 0.0000 |  |
|  |  | GDP | 0.0001 | 0.0000 | 4.9910 | 0.0000 |  |
|  |  | water_prop | 2.4990 | 3.7400 | 0.6680 | 0.5090 | 0.8254 |
|  |  | GDP:water_prop | 0.0000 | 0.0001 | -0.3000 | 0.7660 | 0.9118 |
| wedding | landscape | (Intercept) | 4.7260 | 0.2730 | 17.3110 | <2e-16 |  |
|  |  | GDP | 0.0000 | 0.0000 | 6.5530 | 0.0000 |  |
|  |  | landscape_prop | 0.0830 | 6.5330 | 0.0130 | 0.9900 | 0.9900 |
|  |  | GDP:landscape_prop | 0.0000 | 0.0002 | 0.2230 | 0.8250 | 0.9167 |
| wedding | plant | (Intercept) | 4.8790 | 0.4959 | 9.8390 | 0.0000 |  |
|  |  | GDP | 0.0000 | 0.0000 | 3.1070 | 0.0042 |  |
|  |  | plant_prop | -0.5162 | 1.5900 | -0.3250 | 0.7478 | 0.9118 |
|  |  | GDP:plant_prop | 0.0000 | 0.0001 | 0.3140 | 0.7559 | 0.9118 |
| wedding | animal | (Intercept) | 4.6660 | 0.2169 | 21.5140 | <2e-16 |  |
|  |  | GDP | 0.0001 | 0.0000 | 8.5520 | 0.0000 |  |
|  |  | animal_prop | 24.4200 | 17.5100 | 1.3950 | 0.1740 | 0.5250 |
|  |  | GDP:animal_prop | -0.0010 | 0.0007 | -1.5620 | 0.1290 | 0.5160 |
| wedding | general | (Intercept) | 4.5400 | 0.3408 | 13.3230 | 0.0000 |  |
|  |  | GDP | 0.0001 | 0.0000 | 5.6260 | 0.0000 |  |
|  |  | general_prop | 3.1220 | 4.4260 | 0.7050 | 0.4860 | 0.8254 |
|  |  | GDP:general_prop | -0.0001 | 0.0001 | -0.5320 | 0.5990 | 0.8809 |
| celebration | water | (Intercept) | 5.0710 | 0.3421 | 14.8220 | <2e-16 |  |
|  |  | GDP | 0.0000 | 0.0000 | 3.0100 | 0.0049 |  |
|  |  | water_prop | -1.1360 | 4.6920 | -0.2420 | 0.8101 | 0.9167 |
|  |  | GDP:water_prop | 0.0001 | 0.0001 | 1.0480 | 0.3020 | 0.6750 |
| celebration | landscape | (Intercept) | 5.3330 | 0.2699 | 19.7630 | <2e-16 |  |
|  |  | GDP | 0.0000 | 0.0000 | 4.4640 | 0.0001 |  |
|  |  | landscape_prop | -12.0400 | 6.6150 | -1.8200 | 0.0775 | 0.5090 |
|  |  | GDP:landscape_prop | 0.0004 | 0.0002 | 1.7560 | 0.0880 | 0.5090 |
| celebration | plant | (Intercept) | 5.0090 | 0.5558 | 9.0120 | 0.0000 |  |
|  |  | GDP | 0.0000 | 0.0000 | 3.1490 | 0.0034 |  |
|  |  | plant_prop | 0.2761 | 2.5160 | 0.1100 | 0.9133 | 0.9614 |
|  |  | GDP:plant_prop | 0.0000 | 0.0001 | -0.6220 | 0.5384 | 0.8283 |
| celebration | animal | (Intercept) | 5.0640 | 0.2726 | 18.5780 | <2e-16 |  |
|  |  | GDP | 0.0000 | 0.0000 | 5.2380 | 0.0000 |  |
|  |  | animal_prop | -2.5050 | 8.7010 | -0.2880 | 0.7750 | 0.9118 |
|  |  | GDP:animal_prop | -0.0002 | 0.0003 | -0.6750 | 0.5040 | 0.8254 |
|  | general | (Intercept) | 5.2660 | 0.2953 | 17.8320 | <2e-16 |  |
|  |  | GDP | 0.0000 | 0.0000 | 3.8930 | 0.0004 |  |
|  |  | general_prop | -4.4020 | 3.6030 | -1.2220 | 0.2302 | 0.5756 |
|  |  | GDP:general_prop | 0.0002 | 0.0001 | 1.5130 | 0.1396 | 0.5227 |
| honeymoon | water | (Intercept) | 3.3830 | 2.3040 | 1.4680 | 0.1920 |  |
|  |  | GDP | 0.0001 | 0.0001 | 1.4510 | 0.1970 |  |
|  |  | water_prop | 3.1800 | 7.9180 | 0.4020 | 0.7020 | 0.9118 |
|  |  | GDP:water_prop | -0.0001 | 0.0002 | -0.4880 | 0.6430 | 0.8809 |
| honeymoon | landscape | (Intercept) | 4.1450 | 0.7236 | 5.7280 | 0.0012 |  |
|  |  | GDP | 0.0001 | 0.0000 | 2.2780 | 0.0630 |  |
|  |  | landscape_prop | 7.0960 | 10.5700 | 0.6720 | 0.5269 | 0.8283 |
|  |  | GDP:landscape_prop | -0.0001 | 0.0003 | -0.5060 | 0.6312 | 0.8809 |
| honeymoon | plant | (Intercept) | -1.7550 | 1.4790 | -1.1870 | 0.2801 |  |
|  |  | GDP | 0.0002 | 0.0000 | 5.2170 | 0.0020 |  |
|  |  | plant_prop | 22.2900 | 5.3640 | 4.1560 | 0.0060 | 0.1362 |
|  |  | GDP:plant_prop | -0.0006 | 0.0002 | -3.7890 | 0.0091 | 0.1362 |
| honeymoon | animal | (Intercept) | 4.3700 | 0.5222 | 8.3690 | 0.0002 |  |
|  |  | GDP | 0.0001 | 0.0000 | 3.9290 | 0.0077 |  |
|  |  | animal_prop | -43.0000 | 26.6900 | -1.6110 | 0.1582 | 0.5250 |
|  |  | GDP:animal_prop | 0.0009 | 0.0007 | 1.3420 | 0.2281 | 0.5756 |
| honeymoon | general | (Intercept) | 2.1500 | 1.3480 | 1.5950 | 0.1619 |  |
|  |  | GDP | 0.0001 | 0.0000 | 2.9470 | 0.0257 |  |
|  |  | general_prop | 9.4130 | 5.6720 | 1.6600 | 0.1481 | 0.5227 |
|  |  | GDP:general_prop | -0.0002 | 0.0002 | -1.4060 | 0.2095 | 0.5714 |
| vacation | water | (Intercept) | 4.5020 | 0.3165 | 14.2220 | <2e-16 |  |
|  |  | GDP | 0.0000 | 0.0000 | 2.8180 | 0.0064 |  |
|  |  | water_prop | 0.9666 | 1.0640 | 0.9080 | 0.3671 | 0.7104 |
|  |  | GDP:water_prop | 0.0000 | 0.0000 | 0.3100 | 0.7573 | 0.9118 |
| vacation | landscape | (Intercept) | 4.9470 | 0.2453 | 20.1690 | <2e-16 |  |
|  |  | GDP | 0.0000 | 0.0000 | 4.7110 | 0.0000 |  |
|  |  | landscape_prop | -0.8331 | 0.9494 | -0.8770 | 0.3840 | 0.7200 |
|  |  | GDP:landscape_prop | 0.0000 | 0.0000 | 1.3710 | 0.1750 | 0.5250 |
| vacation | plant | (Intercept) | 4.1690 | 0.4129 | 10.0970 | 0.0000 |  |
|  |  | GDP | 0.0001 | 0.0000 | 3.0940 | 0.0029 |  |
|  |  | plant_prop | 1.9500 | 1.2140 | 1.6070 | 0.1131 | 0.5160 |
|  |  | GDP:plant_prop | -0.0001 | 0.0001 | -0.9420 | 0.3499 | 0.6998 |
| vacation | animal | (Intercept) | 4.7740 | 0.1693 | 28.1960 | <2e-16 |  |
|  |  | GDP | 0.0001 | 0.0000 | 7.4470 | 0.0000 |  |
|  |  | animal_prop | -0.0680 | 1.4710 | -0.0460 | 0.9633 | 0.9796 |
|  |  | GDP:animal_prop | -0.0002 | 0.0001 | -1.8650 | 0.0668 | 0.5010 |
| vacation | general | (Intercept) | 5.0140 | 0.3642 | 13.7670 | <2e-16 |  |
|  |  | GDP | 0.0000 | 0.0000 | 2.7010 | 0.0088 |  |
|  |  | general_prop | -0.8501 | 1.0560 | -0.8050 | 0.4239 | 0.7708 |
|  |  | GDP:general_prop | 0.0001 | 0.0000 | 1.7040 | 0.0933 | 0.5090 |

Table S4. Social contexts and the tags

| Social context | Tag |
| --- | --- |
| Daily routines | “daily”, “routine” |
| Fun activities | “fun”, “activity” |
| Weddings | “wedding” , “marriage” |
| Celebrations | “celebration” |
| Honeymoons | “honeymoon” |
| vacations | “vacation”, “holiday”, “travel” |

Table S5. Most commonly shown nature labels in nature tagged photographs

| Nature category | Label (% of nature-tagged photographs) |
| --- | --- |
| Water | Water (27.9), sea (13.8), body of water (10.3), river (130.3), water resources (10.0), ocean (9.1), coast (8.5), lake (8.4), and watercourse (7.3) |
| Terrestrial landscape | Mountain (18.8), mountainous landforms (13.6), highland (12.2), hill (10.3), mountain rage (9.9), forest (8.8), grassland (7.9), rock (13.9) |
| Plant | Tree (40.2), plant (34.4), grass (15.2), flower (14.4), vegetation (11.7), leaf (11.1), flowering plant (10.0), woody plant (9.9), branch (9.3), botany (7.5) |
| Animal | Wildlife (18.0), bird (10.4), organism (9.5), beak (9.2), vertebrate (8.4) |
| General terms | Nature (32.5), natural environment (18.2), wilderness (17.8), biome (7.3), natural landscape (37.6), landscape (15.0), nature reserve (13.4), horizon (12.4) |

Table S6. Models results summary (generalized mixed effect models to test the relationship between nature and social contexts, excluding repeated photographs).

| Baseline | Nature category | Fixed effect | Estimate | Std.Error | z value | Pr(>\|z\|) |
| --- | --- | --- | --- | --- | --- | --- |
| daily | water | (Intercept) | -2.376 | 0.090 | -26.414 | <2e-16 |
|  |  | groupb.fun_tag | 1.011 | 0.076 | 13.280 | <2e-16 |
|  |  | groupc1.wedding_tag | -0.102 | 0.101 | -1.010 | 0.313 |
|  |  | groupc2.celebration_tag | -0.098 | 0.097 | -1.011 | 0.312 |
|  |  | groupd1.honeymoon_tag | 1.809 | 0.106 | 17.078 | <2e-16 |
|  |  | groupd2.vacation_tag | 1.742 | 0.074 | 23.504 | <2e-16 |
| daily | landscape | (Intercept) | -3.377 | 0.129 | -26.286 | <2e-16 |
|  |  | groupb.fun_tag | 0.914 | 0.108 | 8.442 | <2e-16 |
|  |  | groupc1.wedding_tag | 0.167 | 0.137 | 1.219 | 0.223 |
|  |  | groupc2.celebration_tag | -0.944 | 0.175 | -5.407 | 0.000 |
|  |  | groupd1.honeymoon_tag | 1.940 | 0.136 | 14.230 | <2e-16 |
|  |  | groupd2.vacation_tag | 2.018 | 0.104 | 19.448 | <2e-16 |
| daily | plant | (Intercept) | -1.294 | 0.055 | -23.577 | <2e-16 |
|  |  | groupb.fun_tag | 0.259 | 0.054 | 4.758 | 0.000 |
|  |  | groupc1.wedding_tag | 0.231 | 0.064 | 3.599 | 0.000 |
|  |  | groupc2.celebration_tag | -0.160 | 0.065 | -2.464 | 0.014 |
|  |  | groupd1.honeymoon_tag | 0.635 | 0.093 | 6.855 | 0.000 |
|  |  | groupd2.vacation_tag | 0.544 | 0.053 | 10.187 | <2e-16 |
| daily | animal | (Intercept) | -3.583 | 0.139 | -25.842 | <2e-16 |
|  |  | groupb.fun_tag | 0.803 | 0.123 | 6.522 | 0.000 |
|  |  | groupc1.wedding_tag | -1.222 | 0.237 | -5.162 | 0.000 |
|  |  | groupc2.celebration_tag | -0.626 | 0.180 | -3.483 | 0.000 |
|  |  | groupd1.honeymoon_tag | 0.605 | 0.199 | 3.042 | 0.002 |
|  |  | groupd2.vacation_tag | 0.421 | 0.127 | 3.305 | 0.001 |
| daily | general | (Intercept) | -2.487 | 0.090 | -27.568 | <2e-16 |
|  |  | groupb.fun_tag | 0.649 | 0.080 | 8.092 | 0.000 |
|  |  | groupc1.wedding_tag | -0.059 | 0.104 | -0.566 | 0.571 |
|  |  | groupc2.celebration_tag | -0.550 | 0.110 | -4.983 | 0.000 |
|  |  | groupd1.honeymoon_tag | 1.820 | 0.108 | 16.832 | <2e-16 |
|  |  | groupd2.vacation_tag | 1.858 | 0.076 | 24.368 | <2e-16 |
| fun | water | (Intercept) | -1.378 | 0.068 | -20.275 | <2e-16 |
|  |  | groupc1.wedding_tag | -1.111 | 0.081 | -13.724 | <2e-16 |
|  |  | groupc2.celebration_tag | -1.106 | 0.075 | -14.804 | <2e-16 |
|  |  | groupd1.honeymoon_tag | 0.800 | 0.087 | 9.221 | <2e-16 |
|  |  | groupd2.vacation_tag | 0.737 | 0.042 | 17.436 | <2e-16 |
| fun | landscape | (Intercept) | -2.478 | 0.096 | -25.866 | <2e-16 |
|  |  | groupc1.wedding_tag | -0.747 | 0.105 | -7.126 | 0.000 |
|  |  | groupc2.celebration_tag | -1.859 | 0.151 | -12.338 | <2e-16 |
|  |  | groupd1.honeymoon_tag | 1.036 | 0.104 | 9.972 | <2e-16 |
|  |  | groupd2.vacation_tag | 1.109 | 0.054 | 20.484 | <2e-16 |
| fun | plant | (Intercept) | -1.063 | 0.043 | -24.534 | <2e-16 |
|  |  | groupc1.wedding_tag | -0.025 | 0.053 | -0.474 | 0.636 |
|  |  | groupc2.celebration_tag | -0.419 | 0.054 | -7.737 | 0.000 |
|  |  | groupd1.honeymoon_tag | 0.388 | 0.085 | 4.538 | 0.000 |
|  |  | groupd2.vacation_tag | 0.294 | 0.040 | 7.377 | 0.000 |
| fun | animal | (Intercept) | -2.755 | 0.098 | -28.051 | <2e-16 |
|  |  | groupc1.wedding_tag | -2.025 | 0.215 | -9.432 | <2e-16 |
|  |  | groupc2.celebration_tag | -1.428 | 0.150 | -9.519 | <2e-16 |
|  |  | groupd1.honeymoon_tag | -0.202 | 0.173 | -1.171 | 0.242 |
|  |  | groupd2.vacation_tag | -0.392 | 0.080 | -4.874 | 0.000 |
| fun | general | (Intercept) | -1.848 | 0.067 | -27.383 | <2e-16 |
|  |  | groupc1.wedding_tag | -0.703 | 0.084 | -8.335 | <2e-16 |
|  |  | groupc2.celebration_tag | -1.197 | 0.092 | -13.011 | <2e-16 |
|  |  | groupd1.honeymoon_tag | 1.181 | 0.090 | 13.192 | <2e-16 |
|  |  | groupd2.vacation_tag | 1.218 | 0.046 | 26.402 | <2e-16 |
| wedding | water | (Intercept) | -13.220 | 0.512 | -25.796 | <2e-16 |
|  |  | groupc2.celebration_tag | 0.044 | 0.557 | 0.079 | 0.937 |
| wedding | landscape | (Intercept) | -3.121 | 0.138 | -22.559 | <2e-16 |
|  |  | groupc2.celebration_tag | -1.078 | 0.173 | -6.229 | 0.000 |
| wedding | plant | (Intercept) | -1.060 | 0.045 | -23.769 | <2e-16 |
|  |  | groupc2.celebration_tag | -0.369 | 0.064 | -5.795 | 0.000 |
| wedding | general | (Intercept) | -2.581 | 0.092 | -27.925 | <2e-16 |
|  |  | groupc2.celebration_tag | -0.441 | 0.114 | -3.862 | 0.000 |
| honeymoon | water | (Intercept) | -0.594 | 0.108 | -5.481 | 0.000 |
|  |  | groupd2.vacation_tag | -0.055 | 0.085 | -0.645 | 0.519 |
| honeymoon | landscape | (Intercept) | -1.475 | 0.136 | -10.813 | <2e-16 |
|  |  | groupd2.vacation_tag | 0.068 | 0.099 | 0.693 | 0.489 |
| honeymoon | plant | (Intercept) | -0.678 | 0.089 | -7.631 | 0.000 |
|  |  | groupd2.vacation_tag | -0.088 | 0.085 | -1.047 | 0.295 |
| honeymoon | general | (Intercept) | -0.666 | 0.104 | -6.425 | 0.000 |
|  |  | groupd2.vacation_tag | 0.042 | 0.086 | 0.495 | 0.620 |
